# Editorial: New ideas in: psychology of aging

**DOI:** 10.3389/fpsyg.2024.1383415

**Published:** 2024-03-12

**Authors:** Xukun Yu, Ella Cohn-Schwartz, Zhiwei Zheng, Shufei Yin, Rui Li

**Affiliations:** ^1^CAS Key Laboratory of Mental Health, Institute of Psychology, Beijing, China; ^2^Department of Psychology, University of Chinese Academy of Sciences, Beijing, China; ^3^Gerontology Program, Department of Epidemiology, Biostatistics, and Community Health Sciences, School of Public Health, Ben-Gurion University, Be'er Sheva, Israel; ^4^Department of Psychology, Faculty of Education, Hubei University, Wuhan, China

**Keywords:** aging, memory, mental health, fall prevention, personality

The field of psychology is currently experiencing a significant increase of interest in aging research, driven by the global aging trend. This includes studies that delve into the mechanisms of cognitive and emotional aging, as well as strategies and intervention tools to improve health and wellbeing. In this Research Topic, we present seven original research articles that offer crucial insights into different aspects of aging. Each article contributes novel advancements, with the aim of enhancing our understanding and paving the way for future developments in aging research.

Memory has consistently held a pivotal position in cognitive aging research. Xie et al. explored the decline of the memory system by focusing on the retrogenesis hypothesis. This hypothesis suggests that cognitive abilities in older adults regress in the opposite sequence compared to children's development. Through their examination of declarative and procedural memory in both young and older adults, they revealed that retrogenesis occurs in the declarative memory system but not in the decline order of the two memory systems. Their findings highlight the intricate and multidimensional nature of memory aging.

Shifting the focus to the age-related positivity effect, Yamamoto and Sugiyama investigated the impact of odors on autobiographical memories. Their results demonstrated that older adults recalled more positive memories, rated them higher, and showed positive correlations between odor emotional characteristics and memory attributes. This indicates that odor-evoked emotions have an influence on autobiographical memory. Their findings contribute to our understanding of the mechanisms underlying memory and olfaction, and suggest the potential benefits of utilizing odors to enhance the wellbeing and cognitive functions of older adults.

Pitluk Barash et al. introduced a novel approach to preventing falls in older adults by combining physical therapy exercise (PTE) with Dance Movement Therapy (DMT). While PTE emphasizes structured balance exercises, PTE + DMT incorporates both structured and open instructions to promote spontaneous movements and emotional expressions. They revealed that balance exercises elicit various emotional responses associated with aging, whereas the PTE + DMT intervention goes beyond that by incorporating elements linked to past memories. This integration enhances positive self-image and self-worth, which is believed to positively impact the overall wellbeing of older adults.

The qualitative study conducted by Ma et al. presents a “motivation” model of couple support for digital technology utilization among rural older couples. This model emphasizes the significance of various factors, such as social norms, benefit-driven motivations, and value satisfaction, in influencing couples' inclination toward embracing digital technology. The authors advocated for policymakers and communities to actively address the digital divide that exists in these rural areas, ensuring that the specific needs and expectations of older individuals are met in terms of technology implementation.

Sun et al. explored the interconnectedness of social isolation, aging attitudes, and loneliness among older adults. Their findings revealed that social isolation positively predicts loneliness, with aging attitudes acting as both mediators and moderators. Specifically, social isolation is associated with loneliness via less positive aging attitudes and more negative aging attitudes. Positive aging attitudes alleviate the impact of social isolation on loneliness, while negative aging attitudes exacerbate it. This suggests that addressing social isolation and promoting positive aging attitudes, while reducing negative ones, can effectively alleviate loneliness among older adults and contribute to improving their psychological wellbeing.

Kang utilized data from The British Household Panel Study to examine the link between personality traits and self-rated health (SRH). Their findings revealed that age moderates the relationship between personality traits (e.g., agreeableness, openness, and conscientiousness) and SRH. Moreover, the associations between specific personality traits and SRH varied across different age groups. Kang suggests that future studies exploring these associations should carefully consider the interactions between age and personality traits to provide a better understanding of their impact on self-rated health.

Resilience is closely associated with the adaptive ability of older adults, providing crucial support to cope with challenges and enhance happiness. Wang et al. translated and adapted The Resilience Scale for the Oldest-Old Age (RSO) into Chinese through a rigorous process. The RSO is a nine-item scale developed in Japan. The Chinese version of the RSO has been validated to have the same factor structure as the original scale, demonstrating good validity and reliability in China. This provides a reliable and practical tool for evaluating the resilience of the oldest-old people in China, holding important clinical and application value.

Collectively, the seven studies in this Research Topic provided valuable insights into memory decline, the impact of odors on autobiographical memories, innovative fall prevention strategies, digital technology motivation in older couples, the connection between social isolation and aging attitudes, the link between personality traits and self-rated health, and the assessment of resilience in the oldest-old population in China. We firmly believe that these studies will significantly contribute to our understanding of the psychological processes associated with aging, thereby inspiring further research in prevention and intervention strategies for promoting the health and wellbeing of older individuals.

## Author contributions

XY: Writing – original draft. EC-S: Writing – review & editing. ZZ: Writing – review & editing. SY: Writing – review & editing. RL: Writing – original draft, Writing – review & editing.

